# Development of a Web-Based Intervention for Addressing Distress in Caregivers of Patients Receiving Stem Cell Transplants: Formative Evaluation With Qualitative Interviews and Focus Groups

**DOI:** 10.2196/resprot.7075

**Published:** 2017-06-22

**Authors:** Nicole Amoyal Pensak, Tanisha Joshi, Teresa Simoneau, Kristin Kilbourn, Alaina Carr, Jean Kutner, Mark L Laudenslager

**Affiliations:** ^1^ Hackensack University Medical Center, Department of Research, Cancer Prevention and Control, John Theurer Cancer Center Hackensack, NJ United States; ^2^ University of Colorado Anschutz Medical Campus Department of Medicine Aurora, CO United States; ^3^ VA Eastern Colorado Healthcare System Golden, CO United States; ^4^ University of Colorado-Denver Denver, CO United States; ^5^ University of Colorado Anschutz Medical Campus Department of Psychiatry Aurora, CO United States

**Keywords:** cancer, caregivers, distress, anxiety, depression, stem cell transplant

## Abstract

**Background:**

Caregivers of cancer patients experience significant burden and distress including depression and anxiety. We previously demonstrated the efficacy of an eight session, in-person, one-on-one stress management intervention to reduce distress in caregivers of patients receiving allogeneic hematopoietic stem cell transplants (allo-HSCT).

**Objective:**

The objective of this study was to adapt and enhance the in-person caregiver stress management intervention to a mobilized website (eg, tablet, smartphone, or computer-based) for self-delivery in order to enhance dissemination to caregiver populations most in need.

**Methods:**

We used an established approach for development of a mhealth intervention, completing the first two research and evaluation steps: Step One: Formative Research (eg, expert and stakeholder review from patients, caregivers, and palliative care experts) and Step Two: Pretesting (eg, Focus Groups and Individual Interviews with caregivers of patients with autologous HSCT (auto-HSCT). Step one included feedback elicited for a mock-up version of Pep-Pal session one from caregiver, patients and clinician stakeholders from a multidisciplinary palliative care team (N=9 caregivers and patient stakeholders and N=20 palliative care experts). Step two included two focus groups (N=6 caregivers) and individual interviews (N=9 caregivers) regarding Pep-Pal’s look and feel, content, acceptability, and potential usability/feasibility. Focus groups and individual interviews were audio-recorded. In addition, individual interviews were transcribed, and applied thematic analysis was conducted in order to gain an in-depth understanding to inform the development and refinement of the mobilized caregiver stress management intervention, Pep-Pal (PsychoEducation and skills for Patient caregivers).

**Results:**

Overall, results were favorable. Pep-Pal was deemed acceptable for caregivers of patients receiving an auto-HSCT. The refined Pep-Pal program consisted of 9 sessions (Introduction to Stress, Stress and the Mind Body Connection, How Thoughts Can Lead to Stress, Coping with Stress, Strategies for Maintaining Energy and Stamina, Coping with Uncertainty, Managing Changing Relationships and Communicating Needs, Getting the Support You Need, and Improving Intimacy) delivered via video instruction through a mobilized website.

**Conclusions:**

Feedback from stakeholder groups, focus groups, and individual interviews provided valuable feedback in key areas that was integrated into the development of Pep-Pal with the goal of enhancing dissemination, engagement, acceptability, and usability.

## Introduction

### Background

According to the 2015 National Alliance for Caregiving and the American Association of Retired Persons Public Policy Institute, there are “43.5 million adults in the United States that have provided unpaid care to an adult or child in the prior 12 months” [1]. Estimates from 2013 suggest that unpaid caregivers provide upward of $470 billion in care to their family members [2]. Additionally, missing work due to caregiving responsibilities leads to an estimated $25 billion in lost productivity [2]. While the impact of caregiving on the economy is significant, the emotional impact on the caregiver in terms of depression, anxiety, and distress is also substantial [2].

Caregivers of patients receiving hematopoietic stem cell transplants (HSCTs) are at risk of experiencing significant distress, including depression and anxiety [3,4], given the significant level of patient care that they need to provide. Patients receiving HSCTs require full-time care during the acute transplant and early post-transplant periods, as they face a compromised immune system and thus increased susceptibility to infections, as well as treatment side effects such as fatigue, insomnia, depression, anxiety, and sexual dysfunction [4-8]. As such, caregivers experience multifactorial stressors associated with caring for HSCT patients, leading to complex psychological outcomes that include depression and anxiety, and they are often reluctant to participate in support services given the extra time needed and the demands of their busy schedules. As a result, they can become “silent patients,” being so overburdened with caregiving responsibilities that they neglect to take care of themselves. Furthermore, when the stress of caregiving becomes chronic, caregivers can be at higher risk of developing health problems, which can lead to poorer care for their loved ones [9]. Given the bidirectional nature of the caregiver-patient relationship, helping caregivers manage their stress not only may have beneficial results for the caregiver but also may improve patient outcomes. Thus, convenient access to brief, evidence-based resources is needed to help caregivers manage the stress associated with caregiving [10].

A recent study showed that a brief in-person stress management intervention was effective in reducing the distress of caregivers of patients receiving allogenic HSCTs (allo-HSCTs) [3]. This intervention provided strategies for enhancing the sense of perceived control over stressors inherent to caregiving, understanding role changes, and improving communication during and after transplantation. Additionally, another study assessed caregiver and patient requirements for interactive health communication applications (IHCAs) targeting long-term conditions as well as their criteria for assessing the quality of different programs [11]. IHCAs combine high-quality health information with interactive components (ie, self-assessment tools, behavior change support, peer support, or decision support) and are largely web based. Participants in the study had a favorable view of the potential for Internet interventions to assist with long-term caregiving [11]. However, based on a recent systematic review of Internet-based interventions for caregivers, there are no evidence-based stress management interventions designed specifically for caregivers of HSCT and cancer patients that can be widely disseminated (eg, through technological platforms) [10]. Despite the paucity of evidence-based technological interventions that aim to aid caregivers in managing their stress, 74% of caregivers report being interested in technology that would help them to manage the emotional stress associated with caregiving [12].

Mobile technologies can provide a convenient mode of dissemination for evidence-based resources for managing the stress of caregiving [13]. In a recent study examining caregivers of patients with cancer, most caregivers had access to technological means (eg, Internet and email), and the majority of the caregivers sampled acknowledged that they would potentially utilize caregiver support tools disseminated via the Internet [13]. For overburdened caregivers, mobile technology can provide a valuable source of support for daily self-care that is accessible in their home environment. Mobile technologies have also been effectively implemented as a stand-alone treatment, which is ideal for caregivers with multiple demands who need to care for patients at home [14-23]. As such, the development of an intervention that can be accessed using popular technologies (eg, smartphones, tablets) provides an innovative way to disseminate reliable, empirically supported treatments to caregivers of medically compromised populations who have limited access to in-person support services. In addition, many interventions require heavy resource utilization. Compared to in-person interventions, mobile technologies require fewer personnel resources to disseminate evidence-based treatments and thus may be more cost effective.

### Evidence-Based Intervention

Cognitive-behavioral stress management (CBSM) is a therapeutic intervention that focuses on teaching cognitive and behavioral strategies for coping with cancer [24]. Studies of patients with breast cancer have demonstrated the efficacy of CBSM, with participants in the CBSM intervention groups reporting increased optimism [24], reductions in symptoms of depression and increased ratings of quality of life [25], and improved psychological adjustment to illness [26]. Our previously reported intervention, PsychoEducation, Paced Respiration and Relaxation (PEPRR), was modeled after CBSM and effectively reduced perceived stress, anxiety, and depression in caregivers 3 months post-transplant [3]. However, as structured, the dissemination of that intervention was limited because it required caregivers to meet with a trained clinician in person, which necessitated travel time and schedule flexibility for appointments. Thus, PEPRR may be inaccessible or unacceptable to caregivers who are most in need, namely, those who are so overburdened with caregiving that they cannot participate in this in-person, evidence-based treatment.

### Objective

The purpose of this study was to conduct formative research (eg, stakeholder groups) and pretesting (eg, focus groups and individual interviews) [27] to gather feedback on the look and feel, content, potential acceptability, anticipated usability, and feasibility of Pep-Pal, a mobilized, adapted version of PEPRR. The formative research was then used to inform the development of the final version of Pep-Pal and will be used to facilitate its dissemination.

## Methods

For the purpose of this study, mock-up videos were models of videos (eg, prototypes) used to demonstrate the functionality of the Pep-Pal program and enable testing of the design with potential users. The mock-up videos were adapted based on previously suggested steps for developing and evaluating mHealth interventions [27]. This study followed these two steps: *Step One*, formative research with expert, patient, and caregiver stakeholder groups to develop a usable prototype to test with caregivers in the next step, and *Step Two*, pretesting of Pep-Pal mock-up videos with focus groups and individual interviews.

### Setting

This study was conducted at the University of Colorado Denver Anschutz Medical Campus.

### Step One: Formative Research With Expert, Patient, and Caregiver Stakeholder Groups

A Web-based animated video production service, GoAnimate, INC, was used to develop an initial prototype video session of Pep-Pal. This prototype session consisted of a brief, 10-minute psychoeducation video about stress and stress management delivered by an animated female character posing as a clinical psychologist with a human narrator. The video included content adapted from the PEPRR manual about understanding stress and how stress is experienced by caregivers as well as a brief description of how to manage stress [3]. The prototype video session ended with an animated caregiver character being guided through a body scan exercise. A variety of background music clips were used to enhance engagement.

We conducted three in-person stakeholder meetings, which included one professional stakeholder group (Group One, n=20) and two patient and caregiver stakeholder groups (Groups Two and Three, n=9 total). The professional stakeholder group was included to gather feedback about the acceptability and impressions of the program as well as the potential feasibility of disseminating the intervention. Institutional review board approval was not required for Step One because no demographic data were collected and because feedback was gathered during regular meetings (convenience sample) with experts, patients, and caregivers. Participants in the professional stakeholder group (Group One) included physicians, nurses, social workers, chaplains, and clinical psychologists from a local academic medical center who had expertise in palliative care. Participants in the other stakeholder groups (Groups Two and Three) included men and women between the ages of 30 and 65 who were caregivers of patients with various conditions, such as HSCT recipients. All stakeholder groups watched a 10-minute video example of a Pep-Pal session and provided open-ended feedback in a discussion format. Stakeholder meetings lasted approximately 60 minutes each.

In line with previous recommendations [27], feedback from the stakeholder groups was categorized into several domains: acceptability, anticipated usability, and feasibility. *Acceptability* was defined as the willingness of a user to use the program for its intended purpose. *Anticipated usability* was defined as the extent to which the intended audience could understand the program and find the program to be useful and meaningful. *Feasibility* was defined as the extent to which the program could be made readily available and implemented with the intended audience. In addition, feedback on the look, feel, and content was gathered to enhance acceptability.

### Step Two: Pretesting of Pep-Pal Mock-Up Videos With Focus Groups and Individual Interviews

The goal of Step Two was to conduct focus groups and individual interviews with caregiver participants to assess the acceptability, anticipated usability, and feasibility of the Pep-Pal mock-up videos. Step Two of this study was approved by the University of Colorado Anschutz Medical Campus Institutional Review Board, and informed consent was obtained from all participants.

#### Procedure

Recruitment and eligibility for the focus groups and individual interviews included convenience sampling of caregivers of patients with auto-HSCTs; these caregivers were referred by the Bone Marrow Transplant Clinic and were recruited by phone. The inclusion criteria required participants to be caregivers of patients receiving auto-HSCTs, to be able to speak and read English, and to be at least 18 years of age. The exclusion criteria for caregivers included an absence of a psychiatric or medical condition preventing participation.

#### Focus Groups

A total of 6 Caucasian women who were spousal caregivers between the ages of 46 and 66 participated in the two focus groups. While 8 caregivers were recruited, 2 were unable to attend the focus groups due to their caregiving responsibilities. Notably, 1 caregiver who did not attend asked, “Is there something I can do online?”, supporting the convenience and accessibility of the intervention.

We conducted two semi-structured focus groups. The focus groups were 60 minutes each and were audiotaped. The caregiver participants in the focus groups watched two mock-up videos and provided opinions regarding the videos’ look and feel, content, acceptability, anticipated usability, and feasibility. The first mock-up video included an introduction to Pep-Pal, which provided instructions about the purpose of the Pep-Pal program and how to best utilize the program. The second mock-up video was entitled “Session One: Introduction to Stress Management” and described introductory coping skills for managing stress.

#### Individual Interviews

Based on the results of the focus groups, nine videos of full-length sessions were developed, covering the following topics: introduction to stress management, stress and the mind-body connection, how our thoughts can lead to stress (cognitive distortions), strategies for maintaining energy and stamina, coping with uncertainty, managing relations and coping with your needs, getting the support you need, and improving intimacy. Each Pep-Pal session was less than 10 minutes. Before conducting the individual interviews, two trained clinicians from the original PEPRR study [3] reviewed the Pep-Pal videos and independently both rated the videos as covering all topics from PEPRR. This step was integral to confirming the integrity of Pep-Pal’s adaptation from an evidence-based intervention that effectively reduced symptoms of anxiety, depression, and perceived stress in caregivers of allo-HSCT patients.

We conducted individual interviews with the caregivers of auto-HSCT patients to gather specific feedback on the nine video sessions as well as the overall Pep-Pal program. Participants completed a semi-structured interview that assessed five primary domains: (1) the look and feel of the video sessions, (2) the content addressed in the video sessions, (3) the anticipated usability of the video sessions, (4) the acceptability of the video sessions, and (5) the feasibility of the overall program. Participants were asked about their overall impressions of the sessions, what they may have found helpful or unhelpful in each session, their level of comfort in navigating through the videos, and whether they would consider watching the videos again to review the skills. They were also prompted to provide suggestions for any topics that needed improvement or needed to be added to better serve their needs. Each participant completed a 90-minute, semi-structured interview that consisted of watching an introductory video as well as three other sessions. The interviews were audiotaped and transcribed. At the beginning of the interview, participants were given the choice to watch the sessions on a laptop, tablet, or smartphone. Overall, 8 participants (8/9, 89%) chose to use a laptop, 0 participants used a smartphone, and 1 participant (1/9, 11%) used a tablet to view the videos. We analyzed the interviews after the entire data collection process was complete. Participants were white spousal caregivers (n=9) with a mean age of 59.3 and were predominantly women (n=7).

#### Data Analysis

Applied thematic analysis, which is a thorough yet inductive qualitative approach, was conducted for each interview to identify themes [27]. The first and second authors (NAP and TJ) independently reviewed the transcripts and discussed the themes emerging from the data. They then applied open coding to the transcripts and developed an initial codebook. After discussing the codebook and agreeing upon the codes, TJ coded all transcripts independently and finally identified broad themes within each domain. These themes were then used to further develop and refine the Pep-Pal program. Data saturation was reached when no new themes emerged regarding the look and feel, acceptability, anticipated usability, and feasibility.

## Results

### Step One: Results of Formative Research With Expert, Patient, and Caregiver Stakeholder Groups

Patients, caregivers, and professional stakeholders provided feedback regarding the look and feel, content, acceptability, anticipated usability, and feasibility of Pep-Pal in order to develop an improved prototype for Step Two. Feedback from stakeholders ranged from *“I love what you are trying to do here to meet the needs of caregivers*” to “*this seems like one more thing for caregivers to do.”* In addition, patients, caregivers, and expert stakeholders wanted more introductory information added to explain the benefits for caregivers. The stakeholders preferred a mix of animated and human delivery of information on-screen. Finally, technical feedback included *“the music is too fast* *”* and suggestions to provide more information on respite and other resources earlier in the intervention program. In response to the feedback, the program was modified to enhance its acceptability and anticipated usability. See Table 1 for a summary of the feedback from the stakeholder group meetings and the changes made to refine the Pep-Pal prototype.

### Refining of Pep-Pal Prototype

Several changes were made to the prototype based on the feedback gathered from the three patient, caregiver, and expert clinician stakeholder meetings. These changes to the prototype then informed the pretesting. The most important addition was the creation of a separate introductory video to orient the caregiver to the purpose of the Pep-Pal program and to how best to utilize Pep-Pal. In terms of content and anticipated usability, instructions were added to the introductory video, explaining that Pep-Pal should not replace any in-person support; rather, Pep-Pal should be used in addition to professional support or in the interim, until further support can be accessed. In the video, caregivers were also encouraged to ask for more support from their providers as needed. To improve the look and feel, specific features of the main animated character in Pep-Pal were changed (eg, different hairstyle and smaller eyes). In addition, more human-delivered content was filmed based on the reported preferences of the stakeholders. In terms of content, the topic of Advance Care Planning was removed because it was deemed to be beyond the scope of this intervention.

Other modifications included changing the background music to have a more relaxing “feel”; slowing the pace of the videos; and adding content, including the recommended “dosage” of Pep-Pal (eg, watch each session at least once, for no more than two sessions per week initially, and then watch the videos in order of personal preference) and content encouraging caregivers to “take care of themselves first.” Major and minor changes resulted in the development of two prototype mock-up videos for pretesting in Step Two: (1) Introduction to Pep-Pal and (2) Introduction to Stress Management.

**Table 1:** Summary of feedback from stakeholder group meetings and changes made (Total N=29).

Theme	Patients and Caregiver Stakeholders (n=9)	Professional Stakeholders (n=20)	Changes Made to Pep-Pal
Look and Feel	Music “too loud” “too fast-paced” “make calmer” Animation “don’t like the animated character’s eyes” Like the mix of human and animation Prefer real person on-screen Scenes Like the “hospital room scenes”	Music “too intense and loud” Visual Displays Change “PEP-PAL” to “Pep-Pal” Change “Mini-PEPS” to “Mini-Peps” Pace “Too fast”	Background music changed to be softer, calmer, and slower Changed main animated character Added more human-delivered content Changed PEP-PAL to Pep-Pal Changed Mini-PEPS to Mini-Peps Slowed down session
Content	Tone Include positive and negative examples of change Tone down negative symptoms Change “stress” to “stress management” to portray a more positive tone Information Include information about “diet, complications, chat forum, grief, book recommendations, and community resources” Provide norms of caregiving challenges “Give more of an introduction” “Shorten list of symptoms” Give caregivers “permission to take care of themselves”	Information Delete long information section State “how to best take care of your loved one” “Encourage caregivers to ask for more support from their providers” Use sponge metaphor to explain self-care Get rid of Advance Care Planning: “too complicated” “Introduce team”	Deleted long information section and negative examples Added encouragement for caregivers to ask for more support from their providers Changed “stress” to “stress management” Took out Advance Care Planning Introduced on-screen care delivery team Added introductory session video Shortened list of symptoms Added content for caregivers to give them “permission to take care of themselves”
Acceptability	“Program will be helpful and convenient” “This seems like one more thing for caregivers to do.”	“The video is great!” “The introduction is really good.” “I love what you are trying to do here to meet the needs of caregivers.”	
Anticipated Usability	Timing of Delivery Deliver video right after diagnosis Offer intervention as early as possible Usage Caregivers can watch the videos in the hospital Seems like “one more thing” Can be used by the patient and caregiver together Caregivers will watch this at different stages	Usage Can be used in a “variety of caregiver populations” (eg, dementia patients, oncology patients in Phase I clinical trials) “Can you provide email reminders?”	Feedback noted for final dissemination
Feasibility		Like that it will be tested in a natural usage setting Define minimum dosage of videos (eg, 75%; 1-2 times per week)	Defined recommended dosage as follows: watch each session at least once, no more than 1-2 sessions per week for first-time views; afterward, can re-watch as many times as wanted

**Table 2:** Summary of major iterations to Pep-Pal after focus groups.

Theme	Focus Group Feedback (N=6)	Iterations Made
Look and Feel	Majority preferred mix of animated (“keeps it light”) and human (“provides credibility”) delivery Pace was too fast Background music was relaxing	Kept a mix of animated and human content delivery Slowed pace Used relaxing music throughout all sessions
Content	“Helps you become aware of what's going on with your body.” “Easy to understand.” “Include more positive examples.”	Kept body scan video and information about bodily sensations Added more positive caregiving examples
Acceptability	“I wish it was available now.” “...could use it in the waiting room”	N/A
Anticipated Usability	“Include more step-by-step instruction in the body scan video.” Include breathing exercises	Added more step-by-step instructions to all videos Added deep-breathing exercise
Feasibility	Would like email reminders to complete the sessions throughout the week “Very convenient.” “A website would be great.”	Noted to include reminders and make sessions available on a website platform to facilitate final program dissemination in the pilot randomized controlled trial (RCT)

### Step Two: Results From Focus Groups and Individual Interviews

#### Focus Groups

A summary of the major feedback from the Step Two focus groups is summarized in Table 2 (below). The feedback from the focus group participants was then integrated into the refinement and development of the nine Pep-Pal sessions to be tested in the individual interviews. The focus group participants were asked a series of questions about the program’s look and feel (eg, about the animated characters, music, length, and pace), anticipated usability (ie, satisfaction with the program and clarity of the instructions), and feasibility (ie, how they would use the program).

#### Individual Interviews

Themes were analyzed across each category for look and feel, content, acceptability, anticipated usability, and feasibility. Table 3 (below) summarizes the major themes and feedback gathered from the individual interviews.

#### Summary of Results From Individual Interviews

Overall, 9 participants, who were caregivers of patients who had undergone auto-HSCTs, participated in the individual semi-structured interviews and provided their thoughts and feedback on the Pep-Pal program. The interviews assessed domains including the look and feel, content, anticipated usability, acceptability, and feasibility. Different themes emerged, and the feedback gathered was further integrated to inform the development of the final version of the Pep-Pal program. A summary of the results of the qualitative interviews and changes implemented in the final version of the Pep-Pal program is provided in Table 4.

**Table 3:** Qualitative thematic analysis.

Themes and Subthemes		Quotes
**Look and feel**		
	Animation versus human	“It’s like you almost didn’t value the material enough. The medium does not match the message...I think you need to value the message more.”
		“I was distracted by some of the animation...It caused me to actually concentrate on the corny animation and lose the thread of what was being said.”
	Distractions	“I was watching more the movement and then the words kind of disappearing rather than listening to what it was–I mean, really ascertaining to what it was talking about.”
**Content**		
	Need for personalized examples	“I was trying to connect it [the session] to caregiving, and I was having trouble connecting this particular one to caregiving. I know you did several times use the word caregiver in this, but for me, I was struggling to connect.”
		“I was wondering, does it [the intervention] go into more specifics about the types of stress that come up? Like specifically when they lose all their body hair or you can’t use their bathroom because the chemicals are in there and that’s dangerous?”
		“And they [caregivers] have a fair amount of stress. And not only the ordinary kind of stresses about ‘How do I maintain a healthy attitude?’ and so on but things like, ‘Should we sell this house and move to assisted living?...Who do I ask for help?’”
	Validating the caregiver experience	“I’ve had people tell me it’s harder to be the caretaker than to be the one with the cancer...while I certainly can’t speak to that because I have not been in the other role, it is a very difficult thing, and so it’s nice to have something for us to help us serve, ‘cause it is a very challenging situation to be in.”
		“I think caregivers ask, ‘Am I the only one who’s having this kind of stress or having this intimacy problem?’ But when you address it like this [the program], it helps because then you’re not afraid to realize that you can talk to somebody because other people are going through it too or it wouldn’t be included in here.”
	Combination of one-on-one support and the program	“One of the things that would be helpful to reiterate during the different components [of the program] is that the caregiver doesn’t have to have all the answers, and if there is something that’s unclear or doesn’t make send or is causing stress, just a reminder to go back to the health care providers.”
		“You sit down in front of it [the program]. You’ve got choices to watch it, stop it, fast-forward it. But you really can’t say, ‘Wait a minute. Could you explain that in more detail?’”
**Usability**		
	No difficulty independently navigating sessions	“I couldn’t really tell whether they were encouraging you to do it now or just to file it away for later”
		“I was kind of confused: like should you push STOP and then go ahead and then make a list right then? Or kind of the directions, like, ‘Okay, if you want to, you can push STOP now and go ahead and make that list or continue on.’”
**Acceptability**		
	Caregivers felt this was an acceptable way to get support	“I would [use the program]. Yeah, I’d feel like it would be really, really helpful.”
	Brevity of the sessions and flexibility	“Sometimes you don’t have the time to do anything more than a ten-minute session...things [in the program] are repeated, and it’s like, ‘Oh yeah, I forgot that, let me go back and look at that one.’”
**Feasibility**		
	Program introduction early on during the diagnosis	“I think maybe [introducing the program] in the beginning [of diagnosis]. But then I think also it needs to be kind of–in the beginning, there is so much overwhelming stuff that’s going on that it would be ignored. So it should be like brought up again in a month and brought up again. And just kind of have it available. ‘Cause I think there’s parts of it that I think–especially the relaxation and breathing stuff–that would be so helpful right initially. But I also think that it would be something that could get filed away on a shelf. But it’s nice ‘cause it’s always there. I mean, it’s very portable, very accessible anytime.”
	The need for the program to not be dependent on the Internet	“I think having it [the program] on an Internet interface would be the really appropriate way to go, but there might be situations where Internet access isn’t that available. You might think about having a separate option where you could download it.”

**Table 4:** Summary of qualitative interview results and iterations to Pep-Pal program to obtain final version.

Theme	Individual Interview Feedback (N=9)	Iterations Made
Look and Feel	Majority preferred human-delivered content Include text on-screen Use simple graphics so as to not distract viewer Use more relaxing and softer music	Final Pep-Pal videos include all human-delivered content conveyed by a variety of human clinicians Used simple text and graphics Changed music to be more relaxing and softer
Content	Include more specific caregiver examples Include suggestions for contacting health care providers	Specific caregiver examples were added throughout each session Actress hired to portray caregiver on-screen and to go through examples in each session Caregivers encouraged to speak with health care providers Information for national support resources provided
Acceptability	Want to be able to go back and watch at any time	Easy access to videos is provided (eg, just click this button to watch again at any time)
Anticipated Usability	Add more instructions to videos (eg, stop, pause, do this activity along with video) Liked that the program was not linear, so could watch sessions in any order Pace was too slow in introductory session Pace was too fast in relaxation exercise video	Instructions added throughout Videos not suggested to be viewed in any specific order, but all videos have to be watched at least once Pace was increased in introductory session Pace was slowed in relaxation exercise video
Feasibility	Want to be able to watch videos anywhere (eg, waiting room, bathroom, during medical appointments) Include weekly email reminders to use Pep-Pal Offer program to caregiver at time of diagnosis	Website must be mobilized to enable access on smartphone, tablet, or laptop Automated weekly email reminders are provided with Pep-Pal

## Discussion

### Principal Results

The results from the stakeholder groups, focus groups, and individual interviews supported the acceptability, anticipated usability, and feasibility of Pep-Pal. Feedback was integrated into the final version of Pep-Pal. We found that the domains of usability, acceptability, and feasibility were strongly related to content; when the content resonated with participants, their ability to use the program, willingness to accept the program, and faith in the feasibility of the program increased.

We integrated feedback from Step One, encompassing formative research with expert, patient, and caregiver stakeholder groups, and Step Two, encompassing pretesting of the Pep-Pal mock-up videos with focus groups and individual interviews, into the final version of the Pep-Pal program, which will be tested in a pilot study in Step Three. This process was suggested by Whittaker et al [26] for the development and evaluation of mHealth interventions. Currently, Pep-Pal is available on a mobilized website (eg, can be viewed on a smartphone, laptop, computer, or tablet) that hosts the full-session videos, Mini-Pep videos, and information about the research team. Mini-Peps are brief, 3-minute exercises (eg, related to relaxation, mood boosting, or relationship enhancement) that can be conveniently accessed if the caregiver does not have time to watch a full-session video. Mini-Peps were included in the full-session videos during the formative development study described here. The website is password protected and available only as part of the ongoing pilot RCT to assess the preliminary estimates of efficacy and obtain further information regarding acceptability, anticipated usability, and feasibility. A total of 60 caregivers of patients with advanced illness will be enrolled in this study.

### Limitations

Several limitations were present. First, the patient and caregiver stakeholders were volunteers as part of research advisory groups and thus may represent participants who are more motivated than hard-to-reach or more vulnerable caregiver populations. Second, the developer of the Pep-Pal intervention coordinated the stakeholder groups, focus groups, and individual interviews, so participants may have been reluctant to provide more critical feedback. Third, all participants were white spousal caregivers, which may limit generalizability. Fourth, due to time constraints, detailed demographic data, including socioeconomic status and education level, were not gathered from all participants, thus further limiting the generalizability of this study. However, future studies will gather detailed demographic information. Fifth, the focus group sample size was smaller than anticipated (n=6 per group), and each group was composed of white women, introducing bias as a consequence of the selection of respondents. Finally, participants could not watch all sessions due to time limitations, and thus, saturation was not reached in terms of content. In the corresponding RCT, the developer of Pep-Pal will not conduct the qualitative interviews, the quantitative assessments will be completed on the Web, participants will be representative of more hard-to-reach caregiver populations, and participants will have access to all videos.

### Future Directions

Pep-Pal is being testing in a pilot RCT with caregivers of patients with advanced illness (eg, patients with illness warranting an HSCT, patients with advanced cancer, and those in Phase I oncology trials) to determine the acceptability and feasibility of and preliminary efficacy estimates for Pep-Pal to reduce symptoms of anxiety, depression, perceived stress, and sexual dysfunction. Ultimately, the goal is to conduct a multi-site efficacy RCT of Pep-Pal with caregivers to facilitate future widespread dissemination of Pep-Pal.

## Abbreviations

Allo-HSCTs: allogeneic hematopoietic stem cell transplants
Auto-HSCTs: autologous hematopoietic stem cell transplants
CBSM: cognitive-behavioral stress management
IHCAs: interactive health communication application
PEPRR: PsychoEducation, Paced Respiration and Relaxation
RCT: randomized controlled trial
