# Correction of: Development of a Web-Based Intervention for Addressing Distress in Caregivers of Patients Receiving Stem Cell Transplants: Formative Evaluation With Qualitative Interviews and Focus Groups

**DOI:** 10.2196/resprot.8322

**Published:** 2017-08-15

**Authors:** Nicole Amoyal Pensak, Tanisha Joshi, Teresa Simoneau, Kristin Kilbourn, Alaina Carr, Jean Kutner, Mark L Laudenslager

**Affiliations:** ^1^ Hackensack University Medical Center, Department of Research, Cancer Prevention and Control, John Theurer Cancer Center Hackensack, NJ United States; ^2^ University of Colorado Anschutz Medical Campus Aurora, CO United States; ^3^ VA Eastern Colorado Healthcare System Golden, CO United States; ^4^ University of Colorado-Denver Denver, CO United States; ^5^ University of Colorado Anschutz Medical Campus Department of Psychiatry Aurora, CO United States

An acknowledgment in the paper by Pensak et al, entitled “Development of a Web-Based Intervention for Addressing Distress in Caregivers of Patients Receiving Stem Cell Transplants: Formative Evaluation With Stakeholder Interviews and Focus Groups” [JMIR Res Protoc 2017;6(6):e120], was inadvertently omitted during typesetting. The following information concerning funding should have appeared: "Acknowledgements: Funded in part by grants from NIA T32AG044296 (JK to NAP) and NCI CA126071 (MLL) and a contract from PCORI CE1304-6208 (MLL)."

In addition, the article's original title has been changed to: “Development of a Web-Based Intervention for Addressing Distress in Caregivers of Patients Receiving Stem Cell Transplants: Formative Evaluation With Qualitative Interviews and Focus Groups.”

The acknowledgment and new title will appear in the online version of the paper on the JMIR website on August 15, 2017, together with the publication of this correction notice. Because this was made after submission to PubMed, the correction notice has been submitted to PubMed. The corrected metadata have also been resubmitted to CrossRef.

